# The impact of the COVID-19 pandemic on perinatal services and maternal mental health in the UK

**DOI:** 10.1192/bjo.2022.632

**Published:** 2023-01-13

**Authors:** Lorraine S. Kasaven, Isabel Raynaud, Maria Jalmbrant, Karen Joash, Benjamin P. Jones

**Affiliations:** Department of Surgery and Cancer, Imperial College London, UK; Cutrale Perioperative and Ageing Group, Imperial College London, UK; and Department of Obstetrics and Gynaecology, Queen Charlottes and Chelsea Hospital, Imperial College NHS Trust, UK; West Middlesex University Hospital, Chelsea and Westminster Hospitals NHS Trust, UK; Sloane Court Clinic, UK; Department of Obstetrics and Gynaecology, Queen Charlottes and Chelsea Hospital, Imperial College NHS Trust, UK; Department of Surgery and Cancer, Imperial College London, UK; and Department of Obstetrics and Gynaecology, Queen Charlottes and Chelsea Hospital, Imperial College NHS Trust, UK

**Keywords:** Perinatal psychiatry, community mental health teams, anxiety disorders, psychosocial interventions, patients

## Abstract

**Background:**

COVID-19 has created many challenges for women in the perinatal phase. This stems from prolonged periods of lockdowns, restricted support networks and media panic, alongside altered healthcare provision.

**Aims:**

We aimed to review the evidence regarding the psychological impact on new and expecting mothers following changes to antenatal and postnatal service provision within the UK throughout the pandemic.

**Method:**

We conducted a narrative literature search of major databases (PubMed, Medline, Google Scholar). The literature was critically reviewed by experts within the field of antenatal and perinatal mental health.

**Results:**

Changes to service provision, including the introduction of telemedicine services, attendance of antenatal appointments without partners or loved ones, and lack of support during the intrapartum period, are associated with increased stress, depression and anxiety. Encouraging women and their partners to engage with aspects of positive psychology through newly introduced digital platforms and virtual service provision has the potential to improve access to holistic care and increase mental well-being. An online course, designed by Imperial College Healthcare NHS Trust in response to changes to service provision, focuses on postnatal recovery inspiration and support for motherhood (PRISM) through a 5-week programme. So far, the course has received positive feedback.

**Conclusions:**

The pandemic has contributed to increased rates of mental illness among pregnant and new mothers in the UK. Although the long-term implications are largely unpredictable, it is important to anticipate increased prevalence and complexity of symptoms, which could be hugely detrimental to an already overburdened National Health Service.

The experiences of pregnancy, birth and new motherhood precipitate many physical and psychological stresses for women. Amid a global pandemic such as COVID-19, external stress and anxiety are likely to exacerbate such challenges, resulting in negative effects on mental health.^1^ This is exemplified by the fact that previous natural disasters and epidemics, including the 2002 SARS outbreak, resulted in an increased prevalence of mental illness among pregnant women and new mothers in the areas affected, in addition to poorer perinatal outcomes, such as low birth weight and prematurity.^2^ At the time of writing, the COVID-19 pandemic has led to more than 22 million cases in the UK, with over 170 000 fatalities.^3^ Relentless news coverage and widespread usage of largely unregulated social media have contributed to global and local anxiety. Guidance from the UK Health Security Agency has aimed to promote safety and prevent further spread of infection. However, the ambiguity of rapidly evolving evidence has instead been associated with confusion and negative psychological effects, including heightened risk perception, fear and anxiety.^4,5^ Although the pandemic has affected individuals to varying degrees, for the majority it has changed daily routines and increased health worries, financial insecurity and bereavement.^1^ Pregnant women are particularly vulnerable and susceptible to such stressors because of uncertainties regarding antenatal care, access to services, fear of contracting the virus, social isolation, reduced physical activity and financial concerns.^6^ Considering more than a quarter of a million babies have been born in the UK since the first lockdown in March 2020,^7^ early evidence from the ‘Babies in Lockdown’ report suggests that the pandemic led to at least 60% of new UK mothers feeling concerned for their mental health, with increased self-reported feelings of anxiety, exhaustion, stress and frustration.^7,8^ A recent systematic review and meta-analyses supported the notion that women are struggling, demonstrating increased levels of anxiety and depression among pregnant women throughout the COVID-19 pandemic, compared with pre-pandemic levels.^9,10^

Perinatal mental health disorders contribute significantly to maternal mortality, as well as adverse neonatal, infant and child outcomes, imposing a large burden on families and society as a whole.^1,11^ It is estimated that 20% of women will develop a mental disorder, such as depression, anxiety or psychosis, in the perinatal period.^1^ The social care costs associated with the lifetime risk of perinatal depression and anxiety are estimated to be £75 728 and £34 840, respectively, per woman in the UK.^12^ For these reasons, in 2016 the UK Government agreed to invest more than £290 million into new specialist perinatal mental health services, to ensure that all women within the UK have access to specialist psychiatric in-patient mother and baby units and community services. This is concordant with the antenatal and postnatal mental health guidelines commissioned by the National Institute for Health and Care Excellence.^13^ Despite recent measures to improve perinatal mental health services, findings from the latest confidential enquiry into maternal deaths and morbidity (conducted by Mothers and Babies: Reducing Risk through Audits and Confidential Enquiries across the UK (MBRRACE-UK)) suggest that the level of risk for certain perinatal mental health conditions, including depression and psychosis, in the postpartum period is often underestimated or misattributed, increasing demand for further individualised specialist perinatal mental health services.^14^ Compounded with the issues presented by COVID-19, these factors are likely to overburden our already resource-limited perinatal mental health services even further post-pandemic.^15^

As we continue to provide services through these unprecedented times, healthcare professionals must remain alert to the effects of changes in service provision and access for their patients, and the potential long-term psychological ramifications these may bring. Despite evidence pertaining to the effects of changes in perinatal care in, for example, Austria, France and Japan, there is currently limited UK-specific evidence.^16–18^ Although a number of prospective cohort studies specifically relating to anxiety, depression and post-traumatic stress disorder are being undertaken by the Perinatal Mental Health and COVID-19 Task Force, the findings are not yet available.^1^ The aim of this study is to provide an overview of the psychological effects of COVID-19 on women and their families, through an exploration of some of the critical alterations that have been made to antenatal and postnatal services throughout the pandemic. This exploration will be considered in two main contexts, discussing the effects on the mother as a patient and the mother within their domestic setting.

## The mother as a patient

The healthcare professional–patient relationship is paramount in providing physical and psychological support to pregnant women. On average, patients attend ten antenatal appointments,^19^ and those with more complex pregnancies or severe mental health problems are often required to attend frequently for specialist assessment and intervention in primary and secondary care settings. The World Health Organization champions several central tenets of effective high-quality perinatal care, including respect, person-centeredness and skill, as outlined in their international guidelines.^20^ As UK maternity services, in line with their worldwide counterparts, have had to adapt rapidly during the pandemic, there has been some concern that globally, altered policies and practices aimed at promoting COVID-19 safety may be ‘based on less than robust evidence’, placing clinicians at odds with previous standards of care.^21^ For this reason, the Royal College of Obstetricians and Gynaecologists (RCOG) has advocated an increased emphasis on establishing rapport and trusting relationships during pregnancy.^22^ The underlying precept of such rapidly evolving guidelines focused on the need to strike a balance between caution and compassion in the drive for ‘COVID security’, which involved, for example, the implementation of telemedicine and personal protective equipment (PPE) in clinics to reduce risks from face-to-face interaction.^23^ Albeit essential, the use of PPE in face-to-face meetings or home visits, which partially or fully obscures the face, can contribute to perceived communication barriers through loss of non-verbal cues. Many patients observe their doctors during consultations and respond to their non-verbal cues, including tone of voice, eye contact and posture. Even subtle aspects of non-verbal communication, such as a simple smile from a clinician, can promote feelings of empathy and compassion, which are deemed integral to developing a relationship built on trust between the doctor and patient.^24^ Effective non-verbal communication often influences how approachable a patient perceives their doctor to be, and thus may determine how much information they will volunteer to the clinician.^25^ This is particularly significant in the context of women voluntarily disclosing sensitive information, such as domestic abuse, whereby rates have more than doubled in the UK since the start of the initial lockdown, compared with the average rate in the past 10 years.^8,26^

Telemedicine interventions that include the use of video calls to provide an effective method of communication without the use of PPE, has also become a valuable tool for UK-based perinatal clinicians. In the context of perinatal mental health, a study of 79 women concluded that consultations delivered via such methods were deemed acceptable and associated with high patient satisfaction rates.^27^ In particular, patient groups with limited access to healthcare services placed a higher value on these methods of communication.^28^ Evidence specific to perinatal mental health services is somewhat limited. However, findings from other psychiatric specialties also reiterate the high degree of emotional sensitivity in verbal and non-verbal communication that is acquired during telemedicine consultations. With this in mind, telepsychiatry assessments are perceived to be ‘as good, if not better’ than face-to-face contact.^29^ Furthermore, telemedicine encourages a sense of security and honesty within certain groups, such as patients with schizophrenia.^29^ This is perhaps because consultations take place within a neutral territory and promote patient self-control, creating an environment where individuals feel less threatened or judged.^13^ This is particularly important considering that studies assessing women with mood disorders have highlighted that a desire to receive non-judgemental care was deemed important when engaging with mental health services.^30^

The use of telemedicine also permits the presence of partners or family members during antenatal consultations. During the peak of the pandemic, RCOG guidance advised women to attend face-to-face appointments and ultrasounds alone, to reduce transmission of the virus. It is important to now consider the potential negative consequences this may have had on women with high-risk pregnancies, particularly as lack of partner support is a risk factor for maternal depressive symptoms.^23,31,32^ Partner support during the antenatal period encourages women to prepare for birth complications, prevents delay in seeking medical advice and treatment, promotes management of realistic expectations and reduces stress from collaborative decision-making.^33^ Women with complex pregnancies, who are expected to attend face-to-face appointments, could have been inadvertently coerced into coping with difficult news and information alone.^8^ Evidence also suggests that pre-pandemic, partners were already feeling frustrated and unsupported by their healthcare professionals.^31^ Therefore, their exclusion from the majority of antenatal care and inability to adequately support their partner may have potentiated these negative feelings, and further strained relationships during an already stressful time. This is supported by the fact that less secure attachment and dissatisfaction with support from partners is associated with postpartum depression and post-traumatic stress.^34^

However, telemedicine can also disadvantage certain groups, as it can introduce barriers to providing quality medical, emotional and psychological support.^7^ For example, women from low socioeconomic backgrounds, who are particularly susceptible to maternal depression,^32^ may be at increased risk if they are unable to access healthcare resources virtually. Challenges such as consistent internet access, ownership of appropriate devices or finding private spaces could be detrimental to quality care. Furthermore, attendance at hospitals provides a safe space for victims of domestic abuse, where women can speak to a healthcare professional and maintain confidentiality and security. Telemedicine limits the opportunity to attend an area of safety, where access to opportunities to disclose dangerous situations is limited. Furthermore, during telemedicine consultations it is difficult to ascertain whether abusers are present in the same room and listening to the consultation, thus inhibiting the woman from disclosing sensitive information. Considering that barriers to service access for women with perinatal mental illness pre-pandemic, included associated perceived stigma, poor awareness of facilities and language and cultural barriers,^35^ there is also concern that these factors are only exacerbated in the virtual space.

Barriers to establishing effective connections or rapport may also encourage women to avoid healthcare facilities altogether, because of the stigma and risk associated with COVID-19. This is exemplified by the findings of a four-fold increase in stillbirth rate (9.31 per 1000 births *v*. 2.38 per 1000 births; *P* = 0.01) from one London hospital during the months of the first imposed lockdown.^36^ Reduced admissions could be attributed to the reluctance of women to attend hospital with episodes of reduced foetal movements, fear of contracting the virus or not wanting to burden the National Health Service (NHS).^36^ A disinclination to seek medical advice may prevent timely diagnoses and treatment, thus increasing frustration in engaging with healthcare services, further exacerbating psychological issues within the perinatal period.

This is important, considering that appropriate access to quality prenatal care is paramount to successful maternal and child healthcare.^37^ It is concerning, therefore, that the ‘Babies in Lockdown’ report found that 43% of the new parents surveyed, ‘were not confident that they could access help with their mental health if required’.^7^ Indeed, another UK-based survey found that ‘almost half of all patients reviewed by a specialist mental health midwife reported their support had stopped because of the pandemic’.^8^

The intrapartum experience of labour and birth also has a significant impact on perinatal mental health outcomes, as emotional distress during this time is a risk factor for postnatal depression.^38^ In a recent study, 42.5% of participants reported having to alter their birth plans in response to the COVID-19 pandemic, the uncertainty of which potentiates anxiety for the women affected.^39^ This is likely associated with the guidance recommending only one birth partner, as opposed to two being allowed pre-pandemic.^8^ Continuous support during labour improves outcomes for both women and infants, including increased rates of spontaneous vaginal deliveries, reduced rates of operative vaginal deliveries or caesarean section, reduced use of regional analgesia, shortened duration of labour and higher Apgar scores.^40^ For this reason, the RCOG advocated the presence of at least a single birth partner during labour. Furthermore, the implementation of infection control measures, including the use of isolation rooms and heavy-duty PPE worn by staff members, may induce feelings of impersonal care, preventing mothers from feeling supported by their healthcare providers.^41^ The psychological impact of such changes, in addition to overall perceived negative experiences or previous frustration with healthcare services during the antenatal period, is likely to exacerbate further alienation and engagement with healthcare services, which could increase the risk of birth-related post-traumatic stress disorder.^42^ Should these feelings extend into the postnatal period, new mothers may not feel empowered to ask for support when needed (e.g. regarding difficulties breastfeeding), causing further undue stress and anxiety.

## The mother at home

Extra-familial social support networks are a crucial protective factor of mental health in the perinatal period.^43^ Social isolation, as imposed by lockdowns, is associated with higher levels of stress and causes significant disruption and breakdown of face-to-face interactions, affecting the ability to form and maintain strong and stable relationships.^5,44^ New mothers were unable to attend mum-and-toddler groups, which often help to facilitate new friendships, combat social isolation, establish a routine during maternity leave and provide informal support and information about the local area. They were also discouraged from visiting or being visited by close family and friends, who may have provided practical and emotional support. As such, there have been fewer opportunities for women to express their worries to supportive friends and family, denying them invaluable coping mechanisms.^45^ Consequently, the risk of depression among women has increased, which will continue to adversely affect perinatal mental health experiences as the pandemic continues.^46,47^

Conversely, there are some notable benefits to lockdowns for new mothers with supportive partners. For those living in an emotionally nurturing environment, reinforcement of positive feedback and consistent appraisal from their partners significantly affects maternal mental health.^34^ In particular, prevention of burnout and sharing of household tasks and childcare protects mothers from feeling overwhelmed.^34^ Women who experience higher levels of relationship conflict in the immediate postpartum period are more likely to experience depressive episodes at 8 weeks’ postpartum.^34^ Furthermore, maternal postpartum depression also increases the risk of paternal depression, resulting in less optimal interactions between the father and child and affecting parent–newborn bonding.^13^ However, when fathers with depression spend medium to high amounts of time with their newborns or infants, it can reduce the adverse long-term effects of maternal depression.^48^ Therefore, consistent presence and support from a partner, potentially increased by lockdowns, can have benefits for maternal mental health and attachment.^8^

Maternal mental health problems cost the UK an estimated £8.1 billion per annual cohort of births, secondary to the increased psychological and developmental disturbances in children.^49^ The majority of these costs are attributed to the adverse effects on children, manifesting as child and adolescent depression, anxiety, behavioural problems and special educational needs.^39^ The psychological turmoil experienced by mothers, such as antenatal depression, is associated with long-term consequences such as impairment of emotional cognition, depression in adolescence, autism and attention-deficit hyperactivity disorder.^50,51^ Furthermore, it affects attachment patterns, which affects the offspring's future relationships and their potential to thrive.^50^ Developing positive bonding experiences and secure emotional attachments between the mother and newborn is imperative in providing protective factors for the mental health of new mothers. It helps reinforce positive self-perception of maternal capabilities and fortifies self-esteem.^52^ Evidence from the ‘Babies in Lockdown’ report identified that 34% of parents described a difference in their infants’ interactions and behaviour during this time.^7^ Additionally, 26% of respondents reported increased crying and clinginess from the infant, deemed to be representative of changes to the dynamics of the parent–infant relationship.^7^ Such behaviours have been identified as a direct response to parental stress. This raises the concern of inadequate bonding between mothers and babies during the pandemic, resulting in poorly formed attachments.^5^ In addition, feelings of poor attachment reduce a new mother's self-confidence, as well as affecting their sleep and self-care patterns, which can be detrimental to their overall mental well-being when neglected.

## Other interventions

**Fig. 1:**
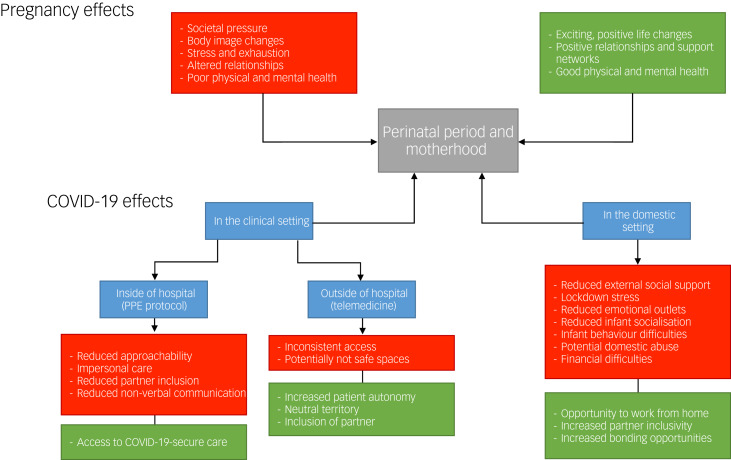
Psychological impact of the COVID-19 pandemic on new and expecting mothers.

As discussed herein, there are a multitude of psychological consequences related to the COVID-19 pandemic in the context of the mother as a patient and the mother in their domestic setting, as summarised in Figure 1. The underlying principles of successful prevention of perinatal mental health is to target appropriate determinants of risk, which often involves individualising patient assessment and treatment. Before the pandemic, various psychological interventions, including cognitive–behavioural therapy courses and interpersonal therapy sessions, proved effective in the management of perinatal depression.^35^ The introduction of preconception mental health counselling into clinics, as opposed to a sole focus on lifestyle and nutritional advice, is also associated with improved perinatal mental health outcomes.^11^ However, many with pre-existing mental health problems have unplanned pregnancies and are therefore not able to access preconception maternity services. For this reason, it is important to have a multidisciplinary approach and liaise closely with our psychiatric colleagues, who should routinely encourage discussions regarding preconception counselling with their patients.^53^

The RCOG, in addition to other support groups, have provided further information for clinicians and patients to ensure that expectant mothers receive the same quality of care that they could have expected before the COVID-19 pandemic. Studies have shown that resilience, despite having some basis in personality, is dynamic and modifiable, and that individual and environmental input can have a significant effect.^54^ Evidence also suggests women have been able to gain resilience through the pandemic by outsourcing from various methods, including virtual communication platforms, engaging in self-care behaviours, practising gratitude and adhering to structures and routines.^55^ Encouraging women and their partners to engage with aspects of positive psychology through newly introduced digital platforms and virtual service provision has the potential to improve access to holistic care and increase well-being.^56^ With this in mind, within our unit at the Imperial College Healthcare NHS Trust, we understood that access to perinatal services may be challenging during the pandemic, and therefore designed an online course focusing on postnatal recovery inspiration and support for motherhood (PRISM).^57^ This consisted of a 5-week online antenatal course where women could register from 28 weeks’ gestation onward and access entirely online. The course aimed to prepare the woman and their partner for birth, with a focus on strategies for improving mental well-being. So far feedback through this method of delivering healthcare and improving patient education has been positive. Other trusts have engaged to adapt information provision for expectant mothers by collaborating, for example, with the Pan-London Perinatal Mental Health Midwifery Forum.^8^ As yet, there has been no formal assessments of the effects of such programmes on pregnant and postnatal women's mental health.

## Conclusions

The COVID-19 pandemic has contributed to increased rates of mental illness among pregnant women and new mothers in the UK. Although the scale of long-term implications is largely unpredictable, it is important to anticipate increased prevalence and complexity of symptoms, which could be hugely detrimental to an already overburdened NHS. The pandemic has already provided an opportunity to encourage lateral thinking when implementing conventional healthcare, with innovative use of technology providing novel and effective assistance to patients and their families. By learning from this pandemic, maternity services within the UK can improve by facilitating prevention, early detection and treatment of perinatal mental health issues, and offering the care that women need and deserve.

## Data Availability

Data availability is not applicable to this article as no new data were created or analysed in this study.
